# Positive psychology and rehabilitation nutrition

**DOI:** 10.1002/jgf2.567

**Published:** 2022-07-09

**Authors:** Hidetaka Wakabayashi

**Affiliations:** ^1^ Department of Rehabilitation Medicine Tokyo Women's Medical University Hospital Tokyo Japan

**Keywords:** goal, PERMA, QOL, strength, well‐being

## Abstract

Rehabilitation nutrition care process includes assessment and diagnostic reasoning, diagnosis, goal setting, intervention, and monitoring. The positive psychology perspective in the rehabilitation nutrition care process may be useful for providing higher quality rehabilitation nutrition.

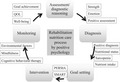

Positive psychology is defined as the scientific study of human thoughts, feelings, and behavior, with a focus on strengths and a better life. Psychological assessment and intervention are necessary in general and family medicine and in rehabilitation nutrition. The relationship between positive psychology and general and family medicine and rehabilitation has been reported.^1^, ^2^ However, positive psychology has not received much attention in these fields until now.

Positive psychology can be useful for maximizing function and quality of life (QOL). Rehabilitation nutrition elicits the highest body functions, activities, participation, and QOL by incorporating both rehabilitation and nutrition.^3^ Intervention to reduce the negative status of negative psychological states, such as depression and anxiety, is important to maximize function and QOL. Depression is a major cause of appetite loss and weight loss in patients requiring rehabilitation nutrition.^4^ Rehabilitation nutrition has been oriented toward reducing the negative status of nutrition and function in clinical practice and clinical research. However, interventions to increase positive psychological status may be useful for eliciting the highest function and QOL, even in the absence of negative psychological status. Regarding QOL, not only health‐related QOL but also non‐health‐related QOL and ikigai (purpose and reason in life) are important for well‐being.

The positive psychology perspective in the rehabilitation nutrition care process may be useful for providing higher quality rehabilitation nutrition (Figure 1). The rehabilitation nutrition care process is a management cycle of rehabilitation nutrition. It consists of five steps, which are assessment and diagnostic reasoning, diagnosis, goal setting, intervention, and monitoring.^3^

**FIGURE 1:**
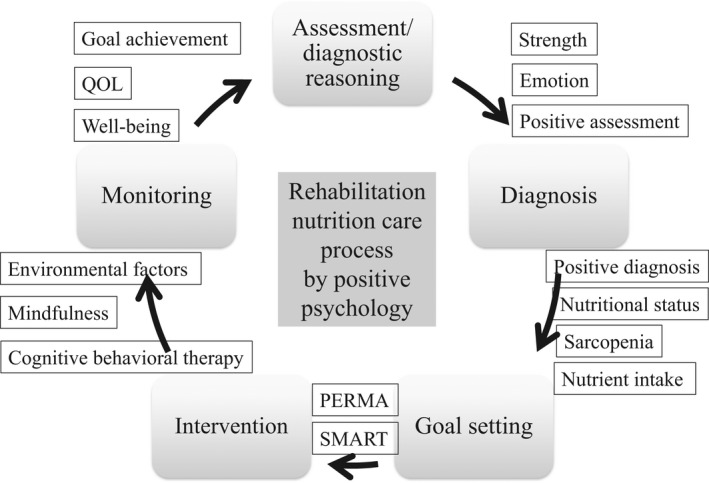
Rehabilitation nutrition care process by positive psychology. Rehabilitation nutrition care process includes assessment and diagnostic reasoning, diagnosis, goal setting, intervention, and monitoring. Both positive and negative assessments and diagnoses are performed. SMART means a goal that is specific, measurable, achievable, realistic/relevant, and timed. PERMA means a goal that is positive emotion, engagement, relationships, meaning, and accomplishment

Rehabilitation nutrition assessment and diagnostic reasoning include a comprehensive assessment by the International Classification of Functioning, Disability and Health (ICF), detailed nutritional assessment, and diagnostic reasoning of malnutrition, sarcopenia, and nutrient intake. Diagnostic reasoning of causes of anorexia, weight loss, and sarcopenia is important in rehabilitation nutrition.^4^ In addition, positive assessment and emotion are important from the perspective of positive psychology. For example, a good appetite is a positive assessment. Moreover, the ICF should be used to assess strengths as well as weaknesses.

Rehabilitation nutrition diagnosis comprises three major categories: nutritional status, sarcopenia, and excess and/or insufficient nutrient intake. The domain of nutritional status includes undernutrition, overnutrition, at risk of undernutrition, at risk of overnutrition, lack of nutrients, and excess of nutrients. Positive diagnosis is important from the perspective of positive psychology. For example, high muscle mass, strong handgrip strength, and fast gait speed are positive diagnoses in patients with sarcopenia.

Rehabilitation nutrition goal setting includes both rehabilitation and nutrition goals. The concept of SMART (Specific, Measurable, Achievable, Realistic/Relevant, and Timed) goal setting should be used in rehabilitation nutrition.^5^ For example, a weight gain of 1 kg in 1 month is a SMART goal. In addition, PERMA (Positive emotion, Engagement, Relationships, Meaning, and Accomplishment) goal setting is important from the perspective of positive psychology.^6^ Positive emotions mean experiencing happiness, gratitude, etc. in the here and now. Engagement means being highly absorbed, immersed, or experiencing flow while engaged in activities. Relationships mean having positive, mutually beneficial relationships with others, characterized by experiences of appreciation. Meaning means the experience of being connected to something larger than the self or serving a larger purpose. Accomplishment means experiencing a sense of mastery over a particular domain or achieving important life/work goals. For example, to be able to perform at piano concerts is a PERMA goal for some people. Setting both SMART and PERMA goals is important.

Rehabilitation nutrition intervention has two aspects, which are “nutrition care management in consideration of rehabilitation” and “rehabilitation in consideration of nutrition.” Psychological interventions, such as mindfulness and cognitive behavioral therapy, are important in rehabilitation nutrition. Moreover, interventions for environmental factors are also important in rehabilitation nutrition. For example, as presented at the 13th Annual Conference of the Japan Primary Care Association in 2022, hospital art classes may be useful for maximizing function and QOL in hospitalized patients.^7^

Rehabilitation nutrition monitoring includes monitoring goal achievement, general condition, nutritional status, nutritional intake, and physical and mental function such as depression, activities of daily living, and social participation. Moreover, well‐being and QOL including ikigai should be monitored from the perspective of positive psychology.

Enhancing the well‐being of healthcare professionals themselves is also important. The number of healthcare professionals who suffer from depression, adjustment disorders, burnout, and other psychological disorders is not small in general and family medicine and rehabilitation nutrition. Any healthcare professional can develop a psychological disorder. Enhancing the well‐being of healthcare professionals themselves by mindfulness seems to be helpful in preventing psychological disorders.^8^ Moreover, a higher well‐being of healthcare professionals may lead to a higher well‐being of patients. The well‐being of healthcare professionals may contribute to maximizing patient function and QOL.

Further clinical research and clinical practice from the perspective of positive psychology are required in general and family medicine and rehabilitation nutrition.

## CONFLICT OF INTEREST

The author declares no conflict of interest for this article.
